# An Overview of Safety and Efficacy Between Hypoxia-Inducible Factor-Prolyl-Hydroxylase Inhibitors and Erythropoietin-Stimulating Agents in Treating Anemia in Chronic Kidney Disease Patients

**DOI:** 10.7759/cureus.42045

**Published:** 2023-07-17

**Authors:** Shamsun Nahar Sonia, Sherie George, Srushti R Shahi, Zahra Ali, Abdelrahman Abaza, Aneeque Jamil, Sai Dheeraj Gutlapalli, Marya Ali, Mrinal J P Oble, Ann Kashmer Yu

**Affiliations:** 1 Internal Medicine, California Institute of Behavioral Neurosciences & Psychology, Fairfield, USA; 2 General Medicine, California Institute of Behavioral Neurosciences & Psychology, Fairfield, USA; 3 Medicine, California Institute of Behavioral Neurosciences & Psychology, Fairfield, USA; 4 Pathology, California Institute of Behavioral Neurosciences & Psychology, Fairfield, USA; 5 Internal Medicine, Richmond University Medical Center, New York, USA; 6 Internal Medicine Clinical Research, California Institute of Behavioral Neurosciences & Psychology, Fairfield, USA; 7 Psychiatry, California Institute of Behavioral Neurosciences & Psychology, Fairfield, USA

**Keywords:** hif-phi, hif-phi safety and efficacy, erythropoietin-stimulating agents, erythropoietin analogs, renal anemia, chronic kidney disease (ckd), ckd

## Abstract

Anemia is one of the common complications in chronic kidney disease (CKD) patients. Erythropoietin and iron deficiencies are the major causes to develop anemia in CKD patients. Untreated anemia is associated with increased morbidity and mortality. Erythropoietin-stimulating agents (ESA) with iron supplementation are the standard for treating renal anemia. Although ESA with iron supplementation is an effective therapy in maintaining serum hemoglobin (Hb) levels, it increases the risk of several life-threatening adverse events such as hypertension, thromboembolism, cardiovascular morbidity, and mortality with long-term use. Therefore, effective alternate therapy with better safety and efficacy is needed to treat renal anemia. The newer oral therapy hypoxia-inducible factor-prolyl-hydroxylase inhibitors (HIF-PHI) can potentially be an effective alternative therapy in treating renal anemia. This review article compares the safety and efficacy between HIF-PHI and ESA in treating anemia in CKD patients.

We conducted a comprehensive literature review of articles, including clinical trials, meta-analyses, and reviews, that compared the safety and efficacy between HIF-PHI and ESA. Studies have shown that the newer oral therapy, HIF-PHI, was non-inferior to ESA to maintain serum Hb levels in CKD patients. Moreover, the adverse event profile was almost similar in both groups. However, as the studies we reviewed have small sample sizes and short duration periods, the long-term effectiveness and safety of HIF-PHI over ESA in treating renal anemia cannot be established.

## Introduction and background

Chronic renal insufficiency or chronic kidney disease (CKD) is one of the major problems worldwide, affecting over 10% global population [1]. CKD is defined as a gradual decline of kidney function over time or persistent signs of kidney damage, such as increased albumin to creatinine ratio or glomerular function rate (GFR) < 60 ml/min/1.73 m2 that persists for at least three months in adults [2]. Anemia, which is defined as hemoglobin (Hb) levels < 12.0 g/dl in females and < 13.0 g/dl in males [3, 4], is a common complication of CKD. Renal anemia occurs in approximately 50% of pre-dialysis patients and over 90% of hemodialysis patients due to impaired erythropoietin production by the damaged kidney [5]. Untreated anemia is associated with increased mortality and morbidity [5]. Current renal anemia management includes erythropoietin-stimulating agents (ESA), iron supplementation, blood transfusion, and hypoxia-inducible factor-prolyl-hydroxylase inhibitors (HIF-PHI) [6].

Recombinant human erythropoietin, ESA, or erythropoietin analogs were introduced in 1989, a milestone in treating anemia in CKD patients [7]. There are several types of erythropoietin analogs. The first generation of ESA, which has a shorter half-life, includes epoetin alfa and epoetin beta; the second and third generation ESA with longer half-life includes darbepoetin alfa and continuous erythropoietin receptor activators, respectively [7]. Although ESA significantly improved anemia management and reduced the need for blood transfusions in CKD patients, it is associated with several adverse effects such as hypertension, thromboembolism, cardiovascular disease, and mortality [8]. In addition, ESA requires adequate intravenous (IV) iron supplementation due to increased iron demand for erythropoiesis [9-11].

HIF-PHI is the newest therapy in managing anemia in CKD patients. By reversibly inhibiting the prolyl-hydroxylase (PHD) enzyme, HIF-PHI stabilizes hypoxia-inducible factors (HIF) activity and regulates endogenous erythropoietin production and iron metabolism [12]. In addition, by suppressing hepatic hepcidin production, HIF-PHIs decrease the need for IV iron supplementation and are associated with fewer cardiovascular risks than ESA [12]. Another advantage of HIF-PHIs is the oral route of administration. The current HIF-PHI formulations include roxadustat, vadadustat, daprodustat, molidustat, and enarodustat [9].

Although ESA is a standard treatment modality for anemia in dialysis-dependent and dialysis-independent CKD patients, its effectiveness is unsatisfactory and not associated with improved clinical outcomes [6]. HIF-PHI could be an effective oral alternative to ESA in treating renal anemia. This literature review aims to compare the safety and efficacy of ESA and HIF-PHIs in treating anemia in individuals with CKD.

## Review

Method

Various keywords, including ESA, HIF-PHI, treatment of renal anemia, and safety and efficacy of HIF-PHI over ESA, were used alone or in combination to find data on two databases, namely, PubMed and Google Scholar. After reviewing the article by the title, abstract, and free full text, we initially selected 129 articles, including clinical trials, meta-analyses, and review articles. We first reviewed articles published only in English. Next, we reviewed relevant free full-text articles and articles with only accessible abstracts. Finally, we selected only 30 relevant articles for the study after thorough screening and reviewing.

Discussion

**Figure 1:**
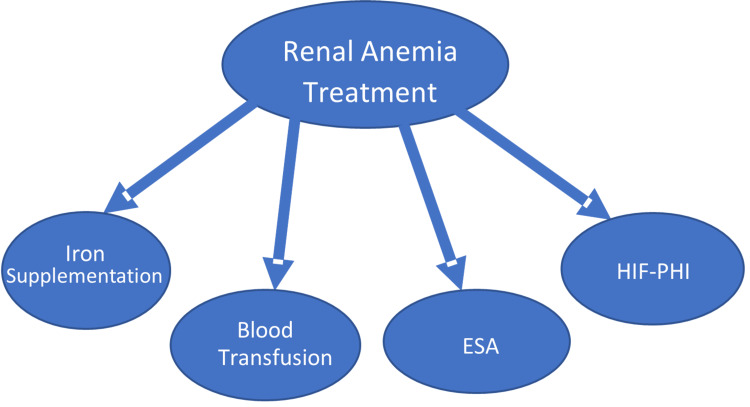
Current Modalities of Treatment in Renal Anemia ESA: Erythropoietin-stimulating agents, HIF-PHI: Hypoxia-inducible factor-prolyl-hydroxylase inhibitors

Major causes of anemia in CKD patients include deficiencies in erythropoietin and iron [13, 14]. Untreated anemia is associated with several adverse outcomes such as fatigue, weakness, breathlessness, light-headedness, cardiovascular disease, cognitive impairment, increased hospitalization, and mortality [9, 13, 15]. Among current regimens of anemia treatment, ESA with iron supplementation is widely used for both dialysis-dependent CKD (DD-CKD) and non-dialysis-dependent CKD (NDD-CKD) patients [16]. Figure 1 shows the current treatment modalities for renal anemia.

Comparison of Mechanism of Action Between ESA and HIF-PHI

Erythropoietin analogs or ESA act like endogenous erythropoietin [17]. By binding to erythropoietin receptors on erythroid progenitor cells, it increases red blood cells (RBC) production and Hb concentration [17-19]. Although ESA corrects anemia effectively in CKD patients and reduces the risk associated with blood transfusion, it increases the need for IV iron supplementation due to the relative increase of iron demand for erythropoiesis [7, 9]. In addition, by increasing blood viscosity, ESA increases the risk of venous thromboembolism, ischemic stroke, myocardial infarction, hypertension, and cardiovascular diseases [17]. Another potential adverse outcome is hyporesponsiveness and resistance to ESA, associated with increased mortality and morbidity, mainly due to functional iron deficiency during inflammation [20]. Furthermore, inflammation upregulates the production of hepcidin from the liver, and its clearance via the kidney also decreases in CKD patients. As a result, hepcidin level increases, which blocks iron mobilization from iron stores and leads to functional iron deficiency [20].

**Figure 2:**
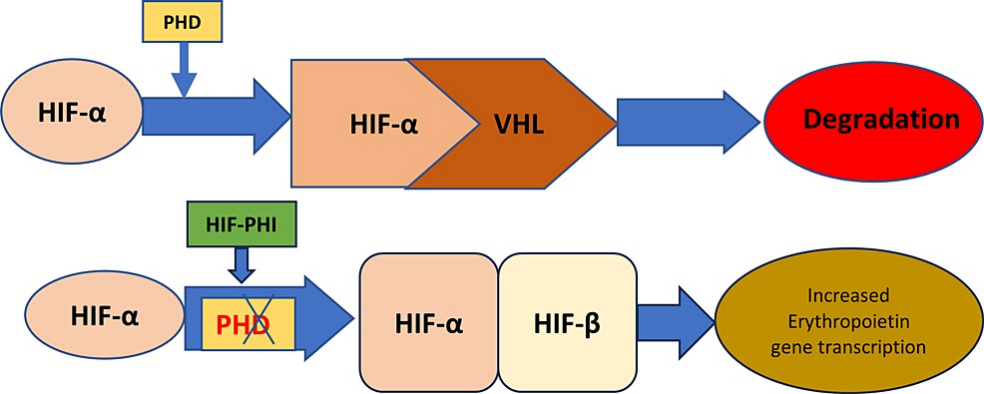
Mechanism of Action of Hypoxia-Inducible Factor-Prolyl-Hydroxylase Inhibitors (HIF-PHI) HIF-α: Hypoxia-inducible factor-alpha, VHL: von HIPPEL-LINDAU protein, PHD: Prolyl-hydroxylase, HIF-PHI: Hypoxia-inducible factor-prolyl-hydroxylase inhibitors, HIF-β: Hypoxia-inducible factor-beta

The use of ESA is associated with an increased risk of cardiovascular morbidity and mortality in CKD patients due to ESA causing a non-physiological increase in erythropoietin [8]. An alternate therapy with better efficacy and fewer adverse effects is needed. The HIF-PHI emerges as one of the newer therapies for treating renal anemia. Erythropoietin synthesis occurs in response to hypoxia and is regulated by HIF. Under normal conditions, hypoxia-inducible factor- alpha (HIF-α) is continuously expressed, but hydroxylated by PHD and then degraded by proteasome ubiquitination after binding to von Hippel-Lindau (VHL) protein [10]. During hypoxia, PHD is inhibited, which allows HIF-α to form a complex with hypoxia-inducible factor-beta (HIF-β), thus increasing the expression of the erythropoietin transcription gene and endogenous erythropoietin production [12]. This pathway also activates multiple target genes related to inflammation, vascular calcification, and angiogenesis [8]. By inhibiting PHD and stabilizing HIF, HIF-PHI increases endogenous erythropoietin production within the physiological range [10]. Figure 2 shows the mechanism of action of HIF-PHI.

Moreover, HIF-PHI improves iron metabolism by increasing intestinal iron absorption, reducing hepcidin levels, and increasing iron mobilization from iron stores [8]. Although it can potentially improve chronic inflammatory status by reducing hepcidin levels and decreasing serum cholesterol levels, its use can increase the risk of vascular calcification, angiogenesis, and tumorigenesis [8].

Comparison of Treatment Efficacy Between ESA and HIF-PHI

ESA is a parenteral formulation used in either subcutaneous or intravenous routes, and it is a breakthrough therapy for treating anemia in CKD patients. For CKD patients, the target Hb level to be corrected with ESA is 11-12 gm/dl [7]. A Hb level > 13 gm/dl is associated with an increased risk of cardiovascular events, morbidity, and mortality [7]. However, the efficacy of ESA is decreased during inflammation due to functional iron deficiency, resulting in hyporesponsiveness and resistance to ESA [20].

On the contrary, the newer oral therapy HIF-PHI improves iron metabolism and acts as an effective therapy irrespective of inflammatory status [8, 21]. Several HIF-PHI, including roxadustat, vadadustat, daprodustat, and molidustat, have been thoroughly studied.

Roxadustat is non-inferior to ESA in maintaining Hb levels between 10 and 12 mg/dl in both DD-CKD and NDD-CKD and reduces cholesterol and hepcidin levels compared to ESA [10, 17, 20, 21]. According to Provenzano et al., roxadustat can maintain Hb levels in hemodialysis patients without iron supplementation in the short term compared to epoetin alpha [21]. Several studies also showed that roxadustat, vadadustat, daprodustat, and molidustat were as effective as ESA in maintaining Hb levels [22-29]. Additionally, they reduced hepcidin levels and increased total iron binding capacity (TIBC) compared to ESA, making them potential oral alternatives to traditional ESA therapy [22-27]. Table 1 compares different clinical studies on the safety and efficacy between HIF-PHI and ESA in treating renal anemia in CKD patients within the previous 10 years. 

**Table 1:** Clinical Studies Comparing the Safety and Efficacy Between HIF-PHI and ESA in the Treatment of Renal Anemia Within the Previous 10 Years. ESA: Erythropoietin-stimulating agents, HIF-PHI: Hypoxia-inducible factor-prolyl-hydroxylase inhibitors, CKD: Chronic kidney disease, Hb: Hemoglobin, vs: Versus

Author & Year of Publication	Drug/Intervention studied	Study Population	Type of Study	Results/Conclusion
Liu et al. [6], 2023	Roxadustat	--	Review	Effective in maintaining Hb levels in CKD patients, but further study is required to determine its long-term safety and efficacy.
Zhang et al. [22], 2021	Roxadustat	--	Meta-analysis	Roxadustat elevated serum Hb levels as ESA, but it may cause hyperkalemia.
Akizawa et al. [23], 2020	Roxadustat versus (vs) Darbepoetin Alfa	Patients with CKD and anemia on hemodialysis in Japan	Phase-3, randomized, double-blind, active-comparator study	Roxadustat was non-inferior to darbepoetin alfa and associated with adverse events consistent with previous reports.
Provenzano et al. [21], 2016	Roxadustat vs Epoetin Alfa	CKD patients with anemia on maintenance hemodialysis	Phase-2, randomized, open-label, active-comparator, safety, and efficacy study	Roxadustat was effective and well-tolerated in maintaining the Hb level.
Agarwal et al. [24], 2022	Vadadustat vs Darbepoetin Alfa	CKD-related anemia patients in phase-3 trials	Pooled data analysis	The rate of adverse events was similar in both groups.
Nangaku et al. [25], 2021	Vadadustat vs Darbepoetin Alfa	Japanese anemic patients on hemodialysis	Phase-3, randomized, double-blind study	Vadadustat was well-tolerated and effectively maintained the Hb level in hemodialysis patients converting from darbepoetin alfa; no serious adverse events in either group.
Haase et al. [26], 2019	Vadadustat vs Epoetin Alfa	Patients on hemodialysis previously treated with ESA	Open-label, phase-2 trial	Vadadustat maintained the Hb level in hemodialysis patients previously treated with epoetin alfa.
Singh et al. [27], 2022	Daprodustat vs Darbepoetin Alfa	CKD patients in incident dialysis	Randomized, prospective, open-label clinical trial	Daprodustat was non-inferior to darbepoetin alfa; it can be a potential oral alternative to ESA.
Akizawa et al. [28], 2020	Daprodustat vs Darbepoetin Alfa	Japanese hemodialysis patients with anemia	Randomized, phase-3, double-blind, active-comparator study	Daprodustat was non-inferior to darbepoetin alpha in Japanese hemodialysis patients switched from ESA.
Akizawa et al. [29], 2019	Molidustat vs Darbepoetin Alfa/Epoetin Alfa	CKD patients with anemia	Randomized, controlled, parallel-group, open-label, multicenter study	Molidustat was well-tolerated and can be an effective alternative to ESA.

Effects on Serum Hepcidin Levels and Iron Metabolism of ESA Versus HIF-PHI

Hepcidin is an acute-phase reactant produced by hepatocytes in the liver during inflammation that regulates iron metabolism and is responsible for functional iron deficiency [14]. Hepcidin prevents iron release from reticuloendothelial macrophages to circulating transferrin and inhibits intestinal iron absorption by targeting, internalization, and degradation of ferroportin [14, 21, 30], resulting in functional iron deficiency. HIF-PHI regulates iron metabolism and reduces hepcidin levels, regardless of inflammation by targeting genes, such as the divalent metal transporter-1 (DMT-1) gene, and others involved in iron metabolism, which ESA cannot [10, 12]. Moreover, ESA is associated with hyporesponsiveness and resistance to maintaining Hb levels due to functional iron deficiency in chronic inflammatory status [20].

Provenzano et al. demonstrated that the reduction of hepcidin level was higher in the roxadustat group than in the epoetin alpha group [21]. According to a meta-analysis by Zhang et al., there was no substantial difference in serum hepcidin levels and serum ferritin levels between roxadustat and ESA groups [22]. However, the transferrin saturation percentage was significantly lower, and the total iron binding capacity was considerably higher in the roxadustat group compared to the ESA group [22]. 

A clinical study regarding the efficacy and safety of vadadustat showed no significant difference in serum ferritin levels, the elevation of the total iron binding capacity, and marginal reduction of serum hepcidin levels at the end of treatment in 52-week treatment period in the vadadustat group compared to the darbepoetin alfa group [25]. Iron-related parameters did not differ significantly among the vadadustat and darbepoetin alfa groups [25]. Another study stated significant changes in iron-related parameters: increased serum TIBC and decreased serum ferritin and hepcidin levels with vadadustat-treated patients previously treated with epoetin alfa [26].

Akizawa et al. demonstrated that daprodustat was associated with less IV iron use, a more significant reduction in serum hepcidin and ferritin levels, and elevation in serum TIBC and serum iron levels compared to darbepoetin alfa, despite achieving similar Hb levels [28]. However, another study showed no significant difference in iron use in daprodustat compared to darbepoetin alfa, although some differences in iron parameters, such as elevation of TIBC with daprodustat and around 17% difference in serum hepcidin level reduction, were observed between the daprodustat and darbepoetin alfa groups [27].

After reviewing all these studies, it is difficult to conclude whether HIF-PHI improves iron metabolism and utilization in renal anemia patients, so further research is needed.

Comparison of Treatment Safety Between ESA and HIF-PHI

The safety profiles of ESA and HIF-PHI were compared, including adverse events, adverse drug reactions, and adverse events of special interest. Adverse events of special interest refer to those related to the treatment of renal anemia with ESA and HIF-PHI, such as cardiovascular events, heart failure, hypertension, retinal disorders, malignancies, hyperkalemia, pulmonary hypertension, and thromboembolism [25].

ESA Versus Roxadustat

According to Liu et al., treatment-emergent adverse events (TEAE) such as nasopharyngitis, vomiting, and hyperkalemia were more frequent with roxadustat than with ESA [6]. Meanwhile, hypertension was more common in the ESA group [6]. Another study indicated that roxadustat significantly reduced mean cholesterol levels, which was not observed with ESA [21]. Adverse events of special interest, such as hypertension, cardiovascular events, and retinal hemorrhage, were similar in both roxadustat and ESA groups [22, 23]. One study reported that the occurrence of retinal hemorrhage between roxadustat and darbepoetin alfa was 3.3% and 3.9%, respectively [23]. A meta-analysis found no significant difference in mortality between the roxadustat and ESA groups [22].

ESA Versus Vadadustat

According to Agarwal, Anand, et al., the safety profile, including treatment-emergent adverse events, TEAEs leading to death, and adverse events of special interest like hypertension, hyperkalemia, congestive heart failure, and malignancies was similar for both vadadustat and ESA groups [24]. The rate of TEAEs, treatment-emergent serious adverse events, and TEAEs leading to death between vadadustat and darbepoetin alfa was 88.9% vs. 89.3%, 58% vs. 59.3%, and 16.1% vs. 16.2% respectively [24]. Other studies suggested no difference in the incidence rate of common adverse events like nasopharyngitis, diarrhea, nausea, and vomiting between vadadustat and ESA [25, 26].

ESA Versus Daprodustat

Common adverse events associated with daprodustat are thromboembolism, hypertension, retinal hemorrhage, and diarrhea [6, 27]. The frequency of adverse event profiles, including adverse events of special interest in daprodustat, was reported to be similar to darbepoetin alfa [27, 28]. Singh et al. reported rates of adverse events of 76% for daprodustat and 72% for darbepoetin alfa, respectively [27].

ESA Versus Molidustat

Akizawa et al. reported that the incidence rate of serious adverse events, such as severe arrhythmias, thromboembolic events, syncope, and heart failure was similar in both molidustat and darbepoetin alpha groups [29]. However, the incidence rate of serious adverse events was higher for molidustat than for epoetin alfa [29].

Limitations

Clinical studies regarding the safety and efficacy of HIF-PHI are currently either in the phase-2 or phase-3 stages. Other concerns include the small sample size and short duration of the study period, making it difficult to draw any conclusions regarding the long-term effectiveness and safety profile of HIF-PHI compared to ESA. Further research with larger populations and longer study periods using multicenter prospective studies is needed to determine the long-term effectiveness of HIF-PHI compared to ESA.

## Conclusions

ESA is the standard of care for CKD patients with anemia, despite being associated with several life-threatening adverse events, such as hypertension, thromboembolism, stroke, myocardial infarction, and mortality, particularly above the target Hb range. HIF-PHI is the newest oral therapy in treating anemia in CKD patients. HIF-PHI can improve iron metabolism in CKD patients, but can increase the risk of vascular calcification, angiogenesis, and tumorigenesis. Several studies have shown that HIF-PHI is as effective as ESA in maintaining serum Hb levels, with a similar adverse events profile. However, due to the small sample sizes and short duration of these studies, we could not draw any conclusions regarding the long-term safety and efficacy of HIF-PHI compared to ESA. More extensive research is necessary to determine the long-term safety and efficacy of HIF-PHI compared to ESA.
